# HER2 Heterogeneity in Personalized Therapy of Gastro-Oesophageal Malignancies: An Overview by Different Methodologies

**DOI:** 10.3390/jpm10010010

**Published:** 2020-02-21

**Authors:** Antonio Ieni, Roberta Cardia, Cristina Pizzimenti, Pio Zeppa, Giovanni Tuccari

**Affiliations:** 1 Department of Human Pathology in Adult and Developmental Age “Gaetano Barresi”, Section of Pathology, University of Messina, 98125 Messina, Italy; robertacardia87@hotmail.it (R.C.); cristina_pizzimenti@virgilio.it (C.P.); tuccari@unime.it (G.T.); 2 Department of Medicine and Surgery, University of Salerno, 84131 Salerno, Italy; pzeppa@unisa.it

**Keywords:** human epidermal growth factor receptor 2 (HER2), immunohistochemistry, dysplasia, gastric cancer, neoplastic heterogeneity, liquid biopsy

## Abstract

Human epidermal growth factor receptor-2 (HER2)-expression gastro-oesophageal adenocarcinomas (GEA) gained interest as an important target for therapy with trastuzumab. In the current review, we focused the current knowledge on HER2 status in dysplastic and neoplastic gastric conditions, analyzing the methodological procedures to identify HER2 expression/amplification, as well as the proposed scoring recommendations. One of the most relevant questions to evaluate the useful impact of HER2 status on therapeutic choice in GEAs is represented by the significant heterogeneity of HER2 protein and gene expression that may affect the targeted treatment selection. Future development of biotechnology will continue to evolve in order to offer more powerful detection systems for the assessment of HER2 status. Finally, liquid biopsy as well as mutation/amplification of several additional genes may furnish an early detection of secondary HER2 resistance mechanisms in GEAs with a better monitoring of the treatment response.

## 1. Introduction

Gastro-oesophageal adenocarcinoma (GEA) represents one of the most aggressive cancers worldwide, in the contest of oesophageal and gastric adenocarcinomas [1]. In the last few decades, oesophageal adenocarcinoma (EA) incidence significantly increased in Western countries, particularly in Caucasian males [2]. This phenomenon seems to be due to an increased occurrence of gastroesophageal reflux disease, complicated by Barrett’s esophagus. Gastric cancer (GC) represents the second cause of neoplastic related mortality, as a result of a combination of environmental factors and numerous genetic alterations [3,4,5,6]. 

Despite some advances in multidisciplinary management, the overall prognosis for patients with GEA remains poor in both sexes, with a worldwide 5-year survival rate lower than 30% for GC and 19% for EA [7]. In recent years, many scientists have performed intensive molecular biology studies to define a genomic classification of GEA [8], leading to a significantly better understanding of new targeted agents acting against specific molecular pathways [8].

The most important molecular innovation comes from a previous randomized phase III trial, also known as trastuzumab for gastric cancer (ToGA) [9]. In this study, a documented predictive progress in terms of overall survival (OS) and median progression-free survival (PFS) has been demonstrated in patients with human epidermal growth factor receptor-2 (HER2) overexpression. These promising results have been achieved by adding trastuzumab to the first-line medical-oncological treatment [9]. Recently, many researchers have investigated the potential prognostic role of HER2 in GEA, even if this topic is still under debate.

Nevertheless, little information is accessible about HER2 function in the GEA carcinogenesis process. However, it is well known that HER2 has been amplified in other several pathological conditions, such as bronchial, breast and cervical dysplasia [10,11,12]. Therefore, we discuss herein the present value of HER2 testing in GE pre-malignant conditions, pre-invasive and invasive GEA, also taking into consideration molecular steps and potential bias.

## 2. Methodological Procedures to Identify Human Epidermal Growth Factor Receptor-2 (HER2) Expression/Amplification

HER2 represents a trans-membrane receptor tyrosine kinase placed on chromosome 17, included in the epidermal growth factor receptor (EGFR) family [13]. As oncogene, its overexpression or amplification has been observed in different neoplastic conditions, leading to unregulated activation of PI3K/AKT/mTOR and MAPK pathways, thus resulting in uncontrolled cell division, proliferation, differentiation and apoptosis [13]. Amplification/overexpression of HER2 has been reported in over 20% of breast cancers and in 9%–38% of patients with GC; in this latter neoplastic entity, its overexpression differs on the basis of histologic type (intestinal versus diffuse) and differentiation (well moderately versus poorly differentiated) [14,15,16,17,18,19]. Currently, the National Comprehensive Cancer Network (NCCN) recommends considering trastuzumab therapy and HER2 assessment by immunohistochemical procedures (IHC) or in situ hybridization (ISH) for patients with advanced, recurrent or metastatic GEA [20]. However, HER2 status can be performed by testing either biopsy or surgical resection specimens [21]. IHC assesses the expression of lateral/basolateral/complete membranous oncoprotein in neoplastic elements, evaluating intensity and percentage of immunoreactive carcinomatous component with scores ranging from 0 to 3+ (Table 1). Therefore, cases scored with 0 or 1+ are considered negative for HER2 expression, while HER2-positive gastric cancer has been defined as IHC 3+; equivocal cases with a score of 2+ should be tested by ISH [20,21,22]. In daily routine practice, a variety of HER2 antibodies are applied, such as HercepTest (Dako, Carpenteria, USA), A0485 (Dako, Carpenteria, USA), 4B5 (Ventana/Roche Tissue Diagnostics, Tucson, USA), CB11 (Novocastra, Newcastle, UK) and SP3 (Thermo Fisher Scientific, Runcorn, UK), as an initial HER2 test. Some studies have shown a considerable variability among different clones of antibodies used to define HER2 expression in GEA [23,24]. In particular, by IHC, it has been reported that the percentage of HER2 overexpression was dissimilar using different antibodies, showing in the antibody A0485 the highest positive rate [25]. Additionally, the same group has revealed that CB11 had the lower sensitivity, but the highest specificity [25]. Moreover, different papers have described that 4B5 and SP3 antibodies had comparable excellent performances, with high negative predictive value (NPV) and higher accuracy compared to the HercepTest, thus suggesting 4B5 and SP3 antibodies are more suitable for first-line tests than the HercepTest in GEA [25]. The ISH methodology includes fluorescence in situ hybridization (FISH), chromogenic in situ hybridization (CISH), silver-enhanced in situ hybridization (SISH) and dual in situ hybridization (DISH); in any case, this procedure plays an important role to detect the presence of gene amplification [26,27,28]. In detail, many different methods use a single HER2 probe to establish the number of HER2 gene copies, but the most powerful recommendation is to use a dual-probe technique, which allows the determination of the HER2 signals ratio to copies of chromosome 17 and HER2 gene copy number [29]. According to the American Society of Clinical Oncology/College of American Pathologists (ASCO/CAP) 2013 guidelines, evaluation of HER2 gene amplification by FISH in GEA is comparable to that applied in breast cancer, in which HER2 amplification has been identified as HER2:CEP17 ratio of ≥2 [30]. Studies about concordance between FISH, CISH and SISH have shown excellent rates of 91%–100%, demonstrating all ISH methodologies as suitable for HER2 amplification testing [30]. More recently, bright field ISH techniques (CISH and SISH) have emerged as preferred assays, since they allow the histopathological assessment (i.e., selecting areas with an intestinal component in GEA), also in archived and indefinitely retrieved specimens, similarly to DISH [30]. Finally, additional studies have compared the performance of DISH relative to FISH and a high degree of concordance between these methods has been achieved [31,32,33]; significantly, none of the cases failed DISH analysis [33].

## 3. HER2 in Gastro-Oesophageal Dysplastic Conditions

It is well known that in the stomach, atrophic gastritis and intestinal metaplasia represent high-risk conditions for gastric cancer development, by the steps of dysplasia and intestinal type gastric adenocarcinoma [34]. Therefore, chronic atrophic gastritis should be diagnosed and graded by pathologists on the basis of the presence of inflammatory elements as well as the decrease of glandular components [35,36]. Currently, the formats for histology reporting called OLGA (operative link for gastritis assessment) and OLGIM (operative link on gastric intestinal metaplasia) systems have been introduced for the staging of gastritis [34,35,36,37]. Additionally, intestinal metaplasia may be reported as “complete” or “incomplete” [38,39,40]. Complete intestinal metaplasia (type I) resembles large bowel morphology composed of the normal small intestinal epithelium with goblet cells and absorptive enterocytes, low immuno expression of typical gastric mucins (such as MUC1, MUC5AC, and MUC6), and MUC2 (intestinal mucin). Conversely, incomplete intestinal metaplasia (type II and type III) is characterized by goblet cells and tortuous/branched crypts with tall columnar elements, in which co-expression of the aforementioned gastric and intestinal mucins has been revealed [38,39,40].

Gastric dysplasia (GD) represents the penultimate step of gastric carcinogenesis, being described as the presence of a histologically unequivocal neoplastic epithelium, without evidence of tissue invasion [41,42]. GD is defined by cellular atypia due to an irregular glandular pattern [41,42]. Nevertheless, some discrepancies between Japanese and European/North American pathologists are emerged in order to categorize GD; in detail, GD has been alternatively called “non-invasive intramucosal carcinoma” or “intraepithelial neoplasia” (IEN) [41,42]. Consequently, to solve this formal linguistic distinction, after the Padova and Vienna international consensus, the World Health Organization (WHO) classification has been proposed (Table 2) [41].

EA is a malignant epithelial carcinoma with glandular differentiation, which develops in the background of Barrett’s esophagus (BE) [43]. It is fundamentally identified as columnar metaplasia which substitutes the stratified squamous epithelium of the distal esophagus [43]. Pathologists play an important role in surveillance of BE patients to identify precursor/dysplastic lesions by morphological assessment as well as discovering patients at high risk [44,45]. In detail, surface maturation, glandular design, cytologic atypia and presence of inflammation and erosions should be clearly identified as relevant morphological characteristics [44,45,46]. Moreover, dysplasia in BE has been categorized into 4 groups on the basis of international consensus [46], similar to the aforementioned WHO classification of gastric dysplasia/intraepithelial neoplasia (Table 3).

Some studies have previously investigated the potential occurrence of HER2 amplification in GE precancerous conditions [47,48,49,50,51,52,53,54]. The first analysis performed in a series of surgical and bioptic gastric samples documented HER2 immunostaining (2+/3+ score) in 12.6% of high-grade (HG)-IEN, while normal gastric mucosa did not stain [50]. It has been demonstrated that HER2 status is conflicting in a paired analysis between dysplasia and invasive GC [50]. Furthermore, HER2 overexpression has been also encountered in low-grade (LG)-IEN, although with significantly lower frequency (4–8%) compared to HG-IEN (16%–20%) [51,52].

A pivotal role for HER2 signaling activation has been shown in EA carcinogenesis and disease progression [55,56,57,58,59]. Interestingly, HER2 overexpression appears to be more common in EA BE-associated than in EA without BE [60]. These data have been confirmed elsewhere, with a higher HER2 immunoexpression rate in esophageal HGD patients (3%–5%), significantly associated to invasive neoplastic component [54]. On the whole, the aforementioned data suggest an early HER2 dysregulation in the oncogenetic progression, both in the stomach and esophagus. Nevertheless, molecular machineries leading to HER2 protein up-regulation remain unclear.

About HER2 protein regulation, specific miRNAs, such as miR-125a-5p, miR-125b, miR-205, miR-331-3p and miR-146a have been proposed to straightly affect HER2 expression [61,62,63,64]. In addition, HER2 transcript suppression may represent the result of miR-125a-5p and/or miR-125b expressed in HER2-positive breast cancer cell lines infected with retroviral constructs [61,62,63]. Moreover, in vitro GC cell lines, a combined treatment with pre–miR-125a-5p and trastuzumab has been demonstrated useful to inhibit cell proliferation and survival [63]. In vivo, patients with GC exhibited an inverse correlation between miR-125a-5p and HER2 status in relation to disease mortality [63]. However, a consistent mutual exclusion of miR-125/ HER2 has been shown in the journey from intestinal metaplasia to LG/HGIEN and adenocarcinoma, suggesting the hypothesis of a progressive dysregulation of the miR125/HER2 loop [63].

## 4. HER2 in Gastro-Oesophageal Malignant Lesions

According to current literature, the frequency of HER2 overexpression/amplification in EA and GC tends to be different, ranging from 7% to 42% [64,65,66,67]. A slight greater HER2 positivity incidence has been reported in EA (24%–35%), more frequently in the subgroup of intestinal-type, in comparison to GC (9.5%–21%) [68,69,70]. It is well known that the rate of HER2 overexpression is strictly related to GC histotype, showing higher frequency in the intestinal histotype (81.6%–91%) in comparison to the diffuse or mixed one (4%–7.9%) [71,72,73,74,75,76,77,78,79]. However, it has been reported an heterogenous pattern of HER2 immunoreactivity in intestinal GEA, exhibiting a combination of HER2-positive and HER2-negative regions [80,81,82,83,84].

**Figure 1:**
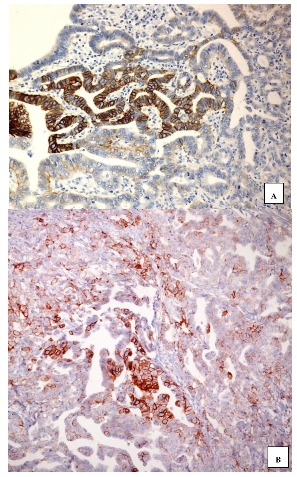
In intestinal-type primary gastric cancer (GC), the heterogeneous 3+ score was present in neoplastic glands either in bioptic ((**A**), 200×) or in surgical specimens, showing immunoreactive elements in direct contact with negative ones ((**B**), 240×) (immunohistochemistry, Mayer’s hemalum counterstain).

The intratumoral heterogeneity of HER2 immunoexpression is particularly significant in GEA, ranging from 4.8% to 50% of cases [85], by contrast with breast carcinomas. Moreover, discordant scores between biopsy and resection specimens may be also attributed to intratumoral heterogeneity of HER2 expression, leading to false-negative results and probable undertreatment [85,86,87,88,89,90]. Additionally, a different HER2 expression between primary cancer and corresponding metastases has also been reported (mean rate 7%), probably related to a clonal selection throughout tumor progression or, alternatively, to intra-tumor HER2 heterogeneity [91,92,93,94,95,96,97,98,99]. This information emphasizes the problem of potential limitations in GEA HER2 analysis on a single specimen (Figure 1A,B), that may not fully be adequate to represent the whole tumor molecular profile [92]. Moreover, during neoplastic progression or therapy, HER2 status could change, making re-evaluation of HER2 status by repeated tissue biopsies really difficult. Therefore, alternative non-invasive approaches have been proposed to monitor the therapeutic efficacy in GEA [99]. Among them, the circulating tumor DNA (ctDNA) has been largely utilized to reveal genetic or epigenetic neoplastic information in clinical practice [99,100]. In particular, ctDNA may be largely used to identify gene mutations as well as variations in gene copy number in the era of next-generation sequencing (NGS) [99]. Consequently, it has been suggested that HER2 amplification could be determined respectively either from ctDNAs or from tumor tissues in GEA patients, by NGS and IHC/DISH [99].

In GEA HER2 status ctDNA testing, also known as ‘liquid biopsy’, may be evaluated by circulating tumor cells (CTCs) and at least one CTC (CTC ≥1) has been detected in 73.5% of GEA samples [101,102,103,104,105,106,107,108]. Consequently, it may be hypothesized that primary HER2-negative tumors may acquire HER2 gene amplification in their CTCs during cancer progression [108]. In our opinion, to improve personalized management strategies in GC, HER2 status of CTCs might be assessed.

It is well known that some patients with HER2-positive GEA, treated with trastuzumab, develop resistance, even if the precise mechanisms are still unexplained [109]. It has been reported that matching pre-treatment and post-progression samples from patients receiving chemotherapy and trastuzumab for advanced HER2-positive GEA, HER2 loss has been encountered in 32% of cases due to a mechanism of resistance [110,111]. Loss of HER2 overexpression might partially explain the failure of second-line anti-HER2 treatment strategies in initially HER2-positive tumors. A continuation of trastuzumab therapy beyond the first progression has been associated with significantly longer median PFS and OS compared with discontinuation, showing clinical benefits in multivariate analysis [112].

Remarkably, the association of different molecular changes has been shown to determine resistance to HER2-targeted treatment in vitro, which can be invalidated by a mutual blockade of HER2 with minor driver mutations [113,114]. In this way, the loss of *PTEN* expression and a low HER2 amplification rate have been associated with primary resistance to trastuzumab-targeted therapy and poor prognosis in HER2-positive GC [115].

Recently, using NGS and whole-exon sequencing techniques, many studies have reported an increased interest in the genomic heterogeneity between primary tumors and metastatic samples in GEA [91,92,93,94,95,96,97,98]. By the aforementioned assays, the detection of numerous gene mutations in known cancer-related genes in GC, such as *TP53, PTEN, ARID1A, APC, CTNNB1, CDH1, PI3KCA* and *KMT2C*, has been performed [116]. In detail, Pectasides et al. have also tried to sequence paired GEA primary and metastatic samples to describe a significant discordance level in genomic alterations, such as *HER2, KRAS, CDK4/6* and *EGFR* [117].

In EA some mutated genes, such as *TP53* (72%), *ELMO1* (25%), *DOCK2* (12%), *CDKN2A* (12%), *ARID1A* (9%), *SMAD4* (8%) and *PIK3CA* (6%), have been identified [102], although no molecular biomarker has been supported in oncological trials. Furthermore, the amplification of *KRAS* (21%), *HER2* (19%), *EGFR* (16%), *CND1* (10%) and *MET* (6%) as well as the loss of *SMAD4* (34%), *CDKN2A* (32%) and *ARID1A* (10%) have been recorded [118,119].

## 5. Conclusions

We fully agree with the clinical impact of HER2 status on therapeutic decision-making in GEAs, even if the significant heterogeneity of HER2 protein and gene expression in GEAs may affect the targeted treatment selection. We can hypothesize that future approaches will determine more accurate methods for HER2 status assessment, making the oncological management of patients more successful. Moreover, data coming from the liquid biopsy as well as mutation/amplification of several genes may help to provide early detection of secondary-resistance mechanisms in GEAs and not only to monitor the treatment response.

## Figures and Tables

**Table 1:** Proposed recommendations for immunohistochemical procedures (IHC) human epidermal growth factor receptor-2 (HER2) testing (modified by College of American Pathologists (CAP), American Society for Clinical Pathology (ASCP) and American Society of Clinical Oncology (ASCO) guidelines on HER2 Testing).

HER2 Score	HER2 IHC in Surgical Specimens	HER2 IHC in Biopsy Specimens	HER2 Assessment
**0**	No membranous reactivity in <10% of neoplastic elements	No membranous reactivity in any cancer cell	Negative
**1+**	Faint or barely membranous immunoreactivity in ≥10% of neoplastic elements	Neoplastic cluster (≥5 neoplastic cells) characterized by a faint or barely membranous immunoreactivity, irrespective of percentage of stained cells	Negative
**2+**	Weak to moderate complete, basolateral or lateral membranous immunoreactivity in ≥10% of neoplastic elements	Neoplastic cluster (≥5 neoplastic elements) characterized by a weak to moderate complete, basolateral or lateral membranous immunoreactivity, irrespective of percentage of stained cells	Equivocal
**3+**	Strong complete, basolateral or lateral membranous immunoreactivity in ≥10% of neoplastic elements	Neoplastic cluster (≥5 neoplastic elements) characterized by a strong complete, basolateral or lateral membranous immunoreactivity, irrespective of percentage of stained cells	Positive

**Table 2:** The World Health Organization (WHO) classification of gastric dysplasia/intraepithelial neoplasia (modified by *WHO Classification of Tumors:* Digestive System Tumors, 5th edition, Lyon: International Agency for Research on Cancer, 2019).

Negative for Intraepithelial Neoplasia/Dysplasia
**Indefinite for intraepithelial neoplasia/dysplasia** (Reactive/regenerative aspect of chronic atrophic gastritis and intestinal metaplasia)
**Low-grade intraepithelial neoplasia/dysplasia/LG-IEN** (Minimal architectural disarray and only mild-to-moderate cytological atypia)
**High-grade intraepithelial neoplasia/dysplasia/HG-IEN** (Prominent architectural disarray, mitoses, high nucleus/cytoplasm ratio and nucleoli)
**Intramucosal invasive neoplasia/intramucosal carcinoma** (Marked glandular crowding, excessive branching and fused/cribriform glands).

**Table 3:** Morphological criteria for dysplasia assessment in BE (modified by *WHO Classification of Tumors:* Digestive System Tumors, 5th edition, Lyon: International Agency for Research on Cancer, 2019).

**Negative for dysplasia** Preserved surface maturation, lack stratification and cytologic atypia is incomplete to the basal part of glands.
**Indefinite for dysplasia (ID)** Modifications in deeper glands indicative but not diagnostic dysplasia with surface maturation and occasional cytologic atypia.
**Low-grade dysplasia (LGD)** Loss of surface maturation and architectural alteration with glandular crowding and mild cytologic atypia.
**High-grade dysplasia (HGD)** Loss of surface maturation and glandular crowding with hyperchromatic nuclei, mitoses and marked cytologic atypia.

## References

[1] Matsuno K., Ishihara R., Ohmori M., Iwagami H., Shichijyo S., Maekawa A., Kanesaka T., Yamamoto S., Takeuchi Y., Higashino K. (2019). Time trends in the incidence of esophageal adenocarcinoma, gastric adenocarcinoma, and superficial esophagogastric junction adenocarcinoma. J. Gastroenterol..

[2] Crane S.J., Richard Locke G., Harmsen W.S., Diehl N.N., Zinsmeister A.R., Joseph Melton L., Romero Y., Talley N.J. (2007). The changing incidence of esophageal and gastric adenocarcinoma by anatomic sub-site. Aliment. Pharm..

[3] Coleman H.G., Xie S.H., Lagergren J. (2018). The Epidemiology of Esophageal Adenocarcinoma. Gastroenterology.

[4] Brown L.M., Devesa S.S., Chow W.H. (2008). Incidence of adenocarcinoma of the esophagus among white Americans by sex, stage, and age. J. Natl. Cancer Inst..

[5] Kim J.J. (2018). Epidemiology of Gastroesophageal Junction Adenocarcinoma in Korea. J. Gastric. Cancer.

[6] Islami F., De Santis C.E., Jemal A. (2019). Incidence Trends of Esophageal and Gastric Cancer Subtypes by Race, Ethnicity, and Age in the United States, 1997-2014. Clin. Gastroenterol. Hepatol..

[7] Natori A., Chan B.A., Sim H.W., Ma L., Yokom D.W., Chen E., Liu G., Darling G., Swallow C., Brar S. (2018). Outcomes by treatment modality in elderly patients with localized gastric and esophageal cancer. Curr. Oncol..

[8] Pasini F., Fraccon A.P., Modena Y., Bencivenga M., Giacopuzzi S., La Russa F., Gusella M., de Manzoni G. (2017). Targeted therapies for advanced and metastatic adenocarcinoma of the gastroesophageal junction: Is there something new?. Gastric. Cancer.

[9] Bang Y.J., Van Cutsem E., Feyereislova A., Chung H.C., Shen L., Sawaki A., Lordick F., Ohtsu A., Omuro Y., Satoh T. (2010). Trastuzumab in combination with chemotherapy versus chemotherapy alone for treatment of HER2-positive advanced gastric or gastro-oesophageal junction cancer (ToGA): A phase 3, open-label, randomised controlled trial. Lancet.

[10] Merrick D.T., Kittelson J., Winterhalder R., Kotantoulas G., Ingeberg S., Keith R.L., Kennedy T.C., Miller Y.E., Franklin W.A., Hirsch F.R. (2006). Analysis of c-ErbB1/epidermal growth factor receptor and c-ErbB2/HER-2 expression in bronchial dysplasia: Evaluation of potential targets for chemoprevention of lung cancer. Clin. Cancer Res..

[11] Lodato R.F., Maguire H.C., Greene M.I., Weiner D.B., LiVolsi V.A. (1990). Immunohistochemical evaluation of c-erbB-2 oncogene expression in ductal carcinoma in situ and atypical ductal hyperplasia of the breast. Mod. Pathol..

[12] Van Bockstal M., Lambein K., Denys H., Braems G., Nuyts A., Van den Broecke R., Cocquyt V., De Wever O., Libbrecht L. (2014). Histopathological characterization of ductal carcinoma in situ (DCIS) of the breast according to HER2 amplification status and molecular subtype. Virchows Archiv..

[13] Yarden Y., Pines G. (2012). The ERBB network: At last, cancer therapy meets systems biology. Nat. Rev. Cancer.

[14] Giuffrè G., Ieni A., Barresi V., Caruso R.A., Tuccari G. (2012). HER2 status in unusual histological variants of gastric adenocarcinomas. J. Clin. Pathol..

[15] Ieni A., Giuffrè G., Lanzafame S., Nuciforo G., Curduman M., Villari L., Roz E., Certo G., Cabibi D., Salomone E. (2012). Morphological and biomolecular characteristics of subcentimetric invasive breast carcinomas in Sicily: A multicentre retrospective study in relation to trastuzumab treatment. Oncol. Lett..

[16] Ieni A., Barresi V., Giuffrè G., Caruso R.A., Lanzafame S., Villari L., Salomone E., Roz E., Cabibi. D., Franco V., Franco V. (2013). HER2 status in advanced gastric carcinoma: A retrospective multicentric analysis from Sicily. Oncol. Lett..

[17] Shan L., Ying J., Lu N. (2013). HER2 expression and relevant clinicopathological features in gastric and gastroesophageal junction adenocarcinoma in a Chinese population. Diagn. Pathol..

[18] Ruggeri R.M., Campennì A., Giuffrè G., Giovanella L., Siracusa M., Simone A., Branca G., Scarfì R., Trimarchi F., Ieni A. (2016). HER2 Analysis in Sporadic Thyroid Cancer of Follicular Cell Origin. Int. J. Mol. Sci..

[19] Gerson J.N., Skariah S., Denlinger C.S., Astsaturov I. (2017). Perspectives of HER2-targeting in gastric and esophageal cancer. Expert Opin. Investig. Drugs.

[20] Rüschoff J., Dietel M., Baretton G., Arbogast S., Walch A., Monges G., Chenard M.P., Penault-Llorca F., Nagelmeier I., Schlake W. (2010). HER2 diagnostics in gastric cancer-guideline validation and development of standardized immunohistochemical testing. Virchows Arch..

[21] Rüschoff J., Hanna W., Bilous M., Hofmann M., Osamura R.Y., Penault-Llorca F., van de Vijver M., Viale G. (2012). HER2 testing in gastric cancer: A practical approach. Mod. Pathol..

[22] Pazo Cid R.A., Anton A. (2013). Advanced HER2-positive gastric cancer: Current and future targeted therapies. Crit. Rev. Oncol. Hematol..

[23] Albarello L., Pecciarini L., Doglioni C. (2011). HER2 testing in gastric cancer. Adv. Anat. Pathol..

[24] Hechtman J.F., Polydorides A.D. (2012). HER2/neu gene amplification and protein overexpression in gastric and gastroesophageal junction adenocarcinoma: A review of histopathology, diagnostic testing, and clinical implications. Arch. Pathol. Lab. Med..

[25] Cho E.Y., Srivastava A., Park K., Kim J., Lee M.H., Do I., Lee J., Kim K.M., Sohn T.S., Kang W.K. (2012). Comparison of four immunohistochemical tests and FISH for measuring HER2 expression in gastric carcinomas. Pathology.

[26] Takehana T., Kunitomo K., Kono K., Kitahara F., Iizuka H., Matsumoto Y., Fujino M.A., Ooi A. (2002). Status of c-erbB-2 in gastric adenocarcinoma: A comparative study of immunohistochemistry, fluorescence in situ hybridization and enzyme-linked immuno-sorbent assay. Int. J. Cancer.

[27] Kim M.A., Jung E.J., Lee H.S., Lee H.E., Jeon Y.K., Yang H.K., Kim W.H. (2007). Evaluation of HER-2 gene status in gastric carcinoma using immunohistochemistry, fluorescence in situ hybridization, and real-time quantitative polymerase chain reaction. Hum. Pathol..

[28] Tsapralis D., Panayiotides I., Peros G., Liakakos T., Karamitopoulou E. (2012). Human epidermal growth factor receptor-2 gene amplification in gastric cancer using tissue microarray technology. World J. Gastroenterol..

[29] Ieni A., Angelico G., Barresi V., Giuffrè G., Arena F., Caruso R.A., Tuccari G. (2018). Human Epidermal Growth Factor Receptor 2 Status in Gastric Carcinomas with Distinctive Prevalent Cribriform Component. Dis. Markers.

[30] Press M.F., Villalobos I., Santiago A., Guzman R., Cervantes M., Gasparyan A., Campeau A., Ma Y., Tsao-Wei D.D., Groshen S. (2016). Assessing the New American Society of Clinical Oncology/College of American Pathologists Guidelines for HER2 Testing by Fluorescence In Situ Hybridization: Experience of an Academic Consultation Practice. Arch. Pathol. Lab. Med..

[31] Mansfield A.S., Sukov W.R., Eckel-Passow J.E., Sakai Y., Walsh F.J., Lonzo M., Wiktor A.E., Dogan A., Jenkins R.B. (2013). Comparison of Fluorescence in Situ Hybridization (FISH) and dual-ISH (DISH) in the Determination of HER2 Status in Breast Cancer. Am. J. Clin. Pathol..

[32] Shao T., Wood M., Wing A., Hnatovska H., Mendes M., Mullen J.B., Chang M.C. (2016). Comparison of HER2 Dual-Color and Fluorescence In Situ Hybridization in Breast Cancer: A Cohort Study Emphasizing Equivocal Cases. Am. J. Clin. Pathol..

[33] Layfield L.J., Wallander M.L., Tripp S.R., Redpath S., Banks P.M. (2017). Comparison of Dual-ISH (DISH) With Fluorescence In Situ Hybridization (FISH) and Correlation With Immunohistochemical Findings for HER2/Neu Status in Breast Carcinoma. Appl. Immunohistochem. Mol. Morphol..

[34] Sugimoto M., Ban H., Ichikawa H., Sahara S., Otsuka T., Inatomi O., Bamba S., Furuta T., Andoh A. (2017). Efficacy of the Kyoto classification of gastritis in identifying patients at high risk for gastric cancer. Intern. Med..

[35] Rugge M., Fassan M., Pizzi M., Farinati F., Sturniolo G.C., Plebani M., Graham D.Y. (2011). Operative link for gastritis assessment vs operative link on intestinal metaplasia assessment. World J. Gastroenterol..

[36] Zhou Y., Li H.-Y., Zhang J.-J., Chen X.-Y., Ge Z.-Z., Li X.-B. (2016). Operative link on gastritis assessment stage is an appropriate predictor of early gastric cancer. World J. Gastroenterol..

[37] Rugge M., Genta R.M. (2005). Staging and grading of chronic gastritis. Hum. Pathol..

[38] Correa P., Piazuelo M.B., Wilson K.T. (2010). Pathology of gastric intestinal metaplasia: Clinical implications. Am. J. Gastroenterol..

[39] Shichijo S., Hirata Y., Niikura R., Hayakawa Y., Yamada A., Ushiku T., Fukayama M., Koike K. (2016). Histological intestinal metaplasia and endoscopic atrophy are predictors of gastric cancer development after Helicobacter pylori eradication. Gastrointest. Endosc..

[40] Gonzalez C.A., Sanz-Anquela J.M., Companioni O., Bonet C., Berdasco M., Lopez C., Mendoza J., Martin-Arranz M.D., Rey E., Poves E. (2016). Incomplete type of intestinal metaplasia has the highest risk to progress to gastric cancer: Results of the spanish follow-up multicenter study. J. Gastroenterol. Hepatol..

[41] Rugge M., Nitti D., Farinati F., di Mario F., Genta R.M. (2005). Non-invasive neoplasia of the stomach. Eur. J. Gastroenterol. Hepatol..

[42] Correa P., Piazuelo M.B. (2012). The gastric precancerous cascade. J. Dig. Dis..

[43] Kuipers E.J., Spaander M.C. (2018). Natural History of Barrett’s Esophagus. Dig. Dis. Sci..

[44] Thrift A.P. (2018). Barrett’s Esophagus and Esophageal Adenocarcinoma: How Common Are They Really?. Dig. Dis. Sci..

[45] Biswas S., Quante M., Leedham S., Jansen M. (2018). The metaplastic mosaic of Barrett’s oesophagus. Virchows. Arch..

[46] Voltaggio L., Montgomery E.A., Lam-Himlin D. (2011). A clinical and histopathologic focus on Barrett esophagus and Barrett-related dysplasia. Arch. Pathol. Lab. Med..

[47] Rossi E., Grisanti S., Villanacci V., Della Casa D., Cengia P., Missale G., Minelli L., Buglione M., Cestari R., Bassotti G. (2009). HER-2 overexpression/amplification in Barrett’s oesophagus predicts early transition from dysplasia to adenocarcinoma: A clinico-pathologic study. J. Cell Mol. Med..

[48] Rossi E., Villanacci V., Bassotti G., Donato F., Festa A., Cengia G., Grisanti S., Cestari R. (2010). TOPOIIalpha and HER-2/neu overexpression/amplification in Barrett’s oesophagus, dysplasia and adenocarcinoma. Histopathology.

[49] Hu Y., Bandla S., Godfrey T.E., Tan D., Luketich J.D., Pennathur A., Qiu X., Hicks D.G., Peters J.H., Zhou Z. (2011). HER2 amplification, overexpression and score criteria in esophageal adenocarcinoma. Mod. Pathol..

[50] Lee S., de Boer W.B., Fermoyle S., Platten M., Kumarasinghe M.P. (2011). Human epidermal growth factor receptor 2 testing in gastric carcinoma: Issues related to heterogeneity in biopsies and resections. Histopathology.

[51] Fassan M., Mastracci L., Grillo F., Zagonel V., Bruno S., Battaglia G., Pitto F., Nitti D., Celiento T., Zaninotto G. (2012). Early HER2 dysregulation in gastric and oesophageal carcinogenesis. Histopathology.

[52] Fusco N., Rocco E.G., Del Conte C., Pellegrini C., Bulfamante G., Di Nuovo F., Romagnoli S., Bosari S. (2013). HER2 in gastric cancer: A digital image analysis in pre-neoplastic, primary and metastatic lesions. Mod. Pathol..

[53] Valente P., Garrido M., Gullo I., Baldaia H., Marques M., Baldaque-Silva F., Lopes J., Carneiro F. (2015). Epithelial dysplasia of the stomach with gastric immunophenotype shows features of biological aggressiveness. Gastric. Cancer..

[54] Ecker B.L., Taylor L., Zhang P.J., Furth E.E., Ginsberg G.G., McMillan M.T., Datta J., Czerniecki B.J., Roses R.E. (2016). HER3 Expression Is a Marker of Tumor Progression in Premalignant Lesions of the Gastroesophageal Junction. PLoS ONE.

[55] Kawaura Y., Tatsuzawa Y., Wakabayashi T., Ikeda N., Matsuda M., Nishihara S. (2001). Immunohistochemical study of p53, c-erbB-2, and PCNA in barrett’s esophagus with dysplasia and adenocarcinoma arising from experimental acid or alkaline reflux model. J. Gastroenterol..

[56] Rygiel A.M., Milano F., Ten Kate F.J., Bergman J.J., Krishnadath K.K. (2010). Low Level of Her-2 Locus Amplification by Fluorescent In Situ Hybridization Does Not Correlate with Her-2 Protein Overexpression by Immunohistochemistry in Barrett’s Esophagus. J. Oncol..

[57] Tanaka T., Fujimura A., Ichimura K., Yanai H., Sato Y., Takata K., Okada H., Kawano S., Tanabe S., Yoshino T. (2012). Clinicopathological characteristics of human epidermal growth factor receptor 2-positive Barrett’s adenocarcinoma. World J. Gastroenterol..

[58] Gowryshankar A., Nagaraja V., Eslick G.D. (2014). HER2 status in Barrett’s esophagus & esophageal cancer: A meta analysis. J. Gastrointest. Oncol..

[59] Realdon S., Dassie E., Fassan M., Dall’Olmo L., Hatem G., Buda A., Arcidiacono D., Diamantis G., Zhang H., Greene M.I. (2015). In vivo molecular imaging of HER2 expression in a rat model of Barrett’s esophagus adenocarcinoma. Dis. Esophagus..

[60] Yoon H.H., Shi Q., Sukov W.R., Wiktor A.E., Khan M., Sattler C.A., Grothey A., Wu T.T., Diasio R.B., Jenkins R.B. (2012). Association of HER2/ErbB2 expression and gene amplification with pathologic features and prognosis in esophageal adenocarcinomas. Clin. Cancer Res..

[61] Scott G.K., Goga A., Bhaumik D., Berger C.E., Sullivan C.S., Benz C.C. (2007). Coordinate suppression of ERBB2 and ERBB3 by enforced expression of micro-RNA miR-125a or miR-125b. J. Biol. Chem..

[62] Nishida N., Mimori K., Fabbri M., Yokobori T., Sudo T., Tanaka F., Shibata K., Ishii H., Doki Y., Mori M. (2011). MicroRNA-125a-5p is an independent prognostic factor in gastric cancer and inhibits the proliferation of human gastric cancer cells in combination with trastuzumab. Clin. Cancer Res..

[63] Fassan M., Pizzi M., Realdon S., Balistreri M., Guzzardo V., Zagonel V., Castoro C., Mastracci L., Farinati F., Nitti D. (2013). The HER2-miR125a5p/miR125b loop in gastric and esophageal carcinogenesis. Hum. Pathol..

[64] Huber A.R., Buscaglia B., Koltz B.R., Henry J., McMahon L., Guo J., Hicks D.G., Whitney-Miller C.L. (2019). Impact of Specimen Type and Specimen Number on HER2 Status in Gastroesophageal Junction and Gastric Adenocarcinoma. Am. J. Clin. Pathol..

[65] Machlowska J., Maciejewski R., Sitarz R. (2018). The Pattern of Signatures in Gastric Cancer Prognosis. Int. J. Mol. Sci..

[66] Wong N.A.C.S., Amary F., Butler R., Byers R., Gonzalez D., Haynes H.R., Ilyas M., Salto-Tellez M., Taniere P. (2018). HER2 testing of gastro-oesophageal adenocarcinoma: A commentary and guidance document from the Association of Clinical Pathologists Molecular Pathology and Diagnostics Committee. J. Clin. Pathol..

[67] Lei Y.Y., Huang J.Y., Zhao Q.R., Jiang N., Xu H.M., Wang Z.N., Li H.Q., Zhang S.B., Sun Z. (2017). The clinicopathological parameters and prognostic significance of HER2 expression in gastric cancer patients: A meta-analysis of literature. World J. Surg. Oncol..

[68] Ieni A., Barresi V., Rigoli L., Caruso R.A., Tuccari G. (2015). HER2 Status in Premalignant, Early, and Advanced Neoplastic Lesions of the Stomach. Dis. Markers.

[69] Abrahao-Machado L.F., Scapulatempo-Neto C. (2016). HER2 testing in gastric cancer: An update. World J. Gastroenterol..

[70] Vakiani E. (2015). HER2 testing in gastric and gastroesophageal adenocarcinomas. Adv. Anat. Pathol..

[71] Fujimoto M., Matsuzaki I., Nishino M., Iwahashi Y., Warigaya K., Kojima F., Ono K., Murata S.I. (2018). HER2 is frequently overexpressed in hepatoid adenocarcinoma and gastric carcinoma with enteroblastic differentiation: A comparison of 35 cases to 334 gastric carcinomas of other histological types. J. Clin. Pathol..

[72] Bang Y.J. (2012). Advances in the management of HER2-positive advanced gastric and gastroesophageal junction cancer. J. Clin. Gastroenterol..

[73] Jørgensen J.T. (2014). Role of human epidermal growth factor receptor 2 in gastric cancer: Biological and pharmacological aspects. World J. Gastroenterol..

[74] Boku N. (2014). HER2-positive gastric cancer. Gastric. Cancer.

[75] Laboissiere R.S., Buzelin M.A., Balabram D., De Brot M., Nunes C.B., Rocha R.M., Cabral M.M., Gobbi H. (2015). Association between HER2 status in gastric cancer and clinicopathological features: A retrospective study using whole-tissue sections. Bmc Gastroenterol..

[76] Wang Y.K., Chen Z., Yun T., Li C.Y., Jiang B., Lv X.X., Chu G.H., Wang S.N., Yan H., Shi L.F. (2015). Human epidermal growth factor receptor 2 expression in mixed gastric carcinoma. World J. Gastroenterol..

[77] Koopman T., Louwen M., Hage M., Smits M.M., Imholz A.L. (2015). Pathologic diagnostics of HER2 positivity in gastroesophageal adenocarcinoma. Am. J. Clin. Pathol..

[78] Hicks D.G., Whitney-Miller C. (2011). HER2 testing in gastric and gastroesophageal junction cancers: A new therapeutic target and diagnostic challenge. Appl. Immunohistochem. Mol. Morphol..

[79] Hofmann M., Stoss O., Shi D., Büttner R., van de Vijver M., Kim W., Ochiai A., Rüschoff J., Henkel T. (2008). Assessment of a HER2 scoring system for gastric cancer: Results from a validation study. Histopathology.

[80] Tajiri R., Ooi A., Fujimura T., Dobashi Y., Oyama T., Nakamura R., Ikeda H. (2014). Intratumoral heterogeneous amplification of ERBB2 and subclonal genetic diversity in gastric cancers revealed by multiple ligation-dependent probe amplification and fluorescence in situ hybridization. Hum. Pathol..

[81] Treacy A.D., Karamchandani J.R., Streutker C.J., Grin A. (2015). HER2 Genetic Heterogeneity in Gastric Cancer: Evaluation According to the College of American Pathologists Breast Cancer Criteria. Appl. Immunohistochem. Mol. Morphol..

[82] Fusco N., Bosari S. (2016). HER2 aberrations and heterogeneity in cancers of the digestive system: Implications for pathologists and gastroenterologists. S. World J. Gastroenterol..

[83] Grillo F., Fassan M., Sarocchi F., Fiocca R., Mastracci L. (2016). HER2 heterogeneity in gastric/gastroesophageal cancers: From benchside to practice. World J. Gastroenterol..

[84] Fazlollahi L., Remotti H.E., Iuga A., Yang H.M., Lagana S.M., Sepulveda A.R. (2018). HER2 Heterogeneity in Gastroesophageal Cancer Detected by Testing Biopsy and Resection Specimens. Arch. Pathol. Lab. Med..

[85] Ieni A., Cardia R., Lentini M., Tuccari G. (2019). Intratumoral HER2 heterogeneity in early gastric carcinomas: Potential bias in therapeutic management. Virchows. Arch..

[86] Yagi S., Wakatsuki T., Yamamoto N., Chin K., Takahari D., Ogura M., Ichimura T., Nakayama I., Osumi H., Shinozaki E. (2019). Clinical significance of intratumoral HER2 heterogeneity on trastuzumab efficacy using endoscopic biopsy specimens in patients with advanced HER2 positive gastric cancer. Gastric. Cancer.

[87] Ieni A., Angelico G., Giuffrè G., Tuccari G. (2018). Discordance Rate of HER2 Status in Primary Gastric Cancer and Synchronous Lymph Node Metastases: Its Impact on Therapeutic Decision and Clinical Management. Pathol. Oncol. Res..

[88] Wakatsuki T., Yamamoto N., Sano T., Chin K., Kawachi H., Takahari D., Ogura M., Ichimura T., Nakayama I., Osumi H. (2018). Clinical impact of intratumoral HER2 heterogeneity on trastuzumab efficacy in patients with HER2-positive gastric cancer. J. Gastroenterol..

[89] Ieni A., Angelico G., Zeppa P., Tuccari G. (2017). Letter to the Editor regarding the paper by Park et al., Extra-gain of HER2-positive cases through HER2 reassessment in primary and metastatic sites in advanced gastric cancer with initially HER2-negative primary tumours: Results of GASTric cancer HER2 reassessment study 1 (GASTHER1). Eur. J. Cancer.

[90] Yoon S.H., Kim Y.H., Lee Y.J., Park J., Kim J.W., Lee H.S., Kim B. (2016). Tumor Heterogeneity in Human Epidermal Growth Factor Receptor 2 (HER2)-Positive Advanced Gastric Cancer Assessed by CT Texture Analysis: Association with Survival after Trastuzumab Treatment. PLoS ONE.

[91] Qiu M.Z., Shi S.M., Chen M., Wang J., Wu Q.N., Sheng H., Zhang H.Z., Yun J.P., Zhou Z.W., Wang F.H. (2017). Comparison of HER2 and Lauren Classification between Biopsy and Surgical Resection Samples, Primary and Metastatic Samples of Gastric Cancer. J. Cancer.

[92] Creemers A., Ter Veer E., de Waal L., Lodder P., Hooijer G.K.J., van Grieken N.C.T., Bijlsma M.F., Meijer S.L., van Oijen M.G.H., van Laarhoven H.W.M. (2017). Discordance in HER2 Status in Gastro-esophageal Adenocarcinomas: A Systematic Review and Meta-analysis. Sci. Rep..

[93] Hedner C., Tran L., Borg D., Nodin B., Jirström K., Eberhard J. (2016). Discordant human epidermal growth factor receptor 2 overexpression in primary and metastatic upper gastrointestinal adenocarcinoma signifies poor prognosis. Histopathology.

[94] Ieni A., Barresi V., Caltabiano R., Caleo A., Bonetti L.R., Lanzafame S., Zeppa P., Caruso R.A., Tuccari G. (2014). Discordance rate of HER2 status in primary gastric carcinomas and synchronous lymph node metastases: A multicenter retrospective analysis. Int. J. Mol. Sci..

[95] Kochi M., Fujii M., Masuda S., Kanamori N., Mihara Y., Funada T., Tamegai H., Watanabe M., Suda H., Takayama T. (2013). Differing deregulation of HER2 in primary gastric cancer and synchronous related metastatic lymph nodes. Diagn. Pathol..

[96] Fassan M., Ludwig K., Pizzi M., Castoro C., Guzzardo V., Balistreri M., Zaninotto G., Ruol A., Giacomelli L., Ancona E. (2012). Human epithelial growth factor receptor 2 (HER2) status in primary and metastatic esophagogastric junction adenocarcinomas. Hum. Pathol..

[97] Kim M.A., Lee H.J., Yang H.K., Bang Y.J., Kim W.H. (2011). Heterogeneous amplification of ERBB2 in primary lesions is responsible for the discordant ERBB2 status of primary and metastatic lesions in gastric carcinoma. Histopathology.

[98] Bozzetti C., Negri F.V., Lagrasta C.A., Crafa P., Bassano C., Tamagnini I., Gardini G., Nizzoli R., Leonardi F., Gasparro D. (2011). Comparison of HER2 status in primary and paired metastatic sites of gastric carcinoma. Br. J. Cancer.

[99] Wang H., Li B., Liu Z., Gong J., Shao L., Ren J., Niu Y., Bo S., Li Z., Lai Y. (2018). HER2 Copy Number of Circulating Tumour DNA Functions as a Biomarker to Predict and Monitor Trastuzumab Efficacy in Advanced Gastric Cancer. Eur. J. Cancer.

[100] Diaz L.A., Bardelli A. (2014). Liquid Biopsies: Genotyping Circulating Tumor DNA. J. Clin. Oncol..

[101] Shoda K., Masuda K., Ichikawa D., Arita T., Miyakami Y., Watanabe M., Konishi H., Imoto I., Otsuji E. (2015). HER2 amplification detected in the circulating DNA of patients with gastric cancer: A retrospective pilot study. Gastric. Cancer.

[102] Shimada H. (2015). Is “liquid biopsy” useful for assessing HER2 status in gastric cancer?. J. Gastroenterol..

[103] Mishima Y., Matsusaka S., Chin K., Mikuniya M., Minowa S., Takayama T., Shibata H., Kuniyoshi R., Ogura M., Terui Y. (2017). Detection of HER2 Amplification in Circulating Tumor Cells of HER2-Negative Gastric Cancer Patients. Target. Oncol..

[104] Shoda K., Ichikawa D., Fujita Y., Masuda K., Hiramoto H., Hamada J., Arita T., Konishi H., Komatsu S., Shiozaki A. (2017). Monitoring the HER2 copy number status in circulating tumor DNA by droplet digital PCR in patients with gastric cancer. Gastric. Cancer.

[105] Uguen A. (2017). About HER2 monitoring using liquid biopsies in patients with gastric cancer. Gastric. Cancer.

[106] Shen L. (2018). Liquid biopsy: A powerful tool to monitor trastuzumab resistance in HER2-positive metastatic gastric cancer. Cancer Commun..

[107] Lee J., Franovic A., Shiotsu Y., Kim S.T., Kim K.M., Banks K.C., Raymond V.M., Lanman R.B. (2019). Detection of *ERBB2* (HER2) Gene Amplification Events in Cell-Free DNA and Response to Anti-HER2 Agents in a Large Asian Cancer Patient Cohort. Front. Oncol..

[108] Iwatsuki M., Toyoshima K., Watanabe M., Hayashi N., Ishimoto T., Eto K., Iwagami S., Baba Y., Yoshida N., Hayashi A. (2013). Frequency of HER2 expression of circulating tumour cells in patients with metastatic or recurrent gastrointestinal cancer. Br. J. Cancer.

[109] Wang D.S., Liu Z.X., Lu Y.X., Bao H., Wu X., Zeng Z.L., Liu Z., Zhao Q., He C.Y., Lu J.H. (2018). Liquid biopsies to track trastuzumab resistance in metastatic HER2-positive gastric cancer. Gut.

[110] Pietrantonio F., Fucà G., Morano F., Gloghini A., Corso S., Aprile G., Perrone F., De Vita F., Tamborini E., Tomasello G. (2018). Biomarkers of Primary Resistance to Trastuzumab in HER2-Positive Metastatic Gastric Cancer Patients: The AMNESIA Case-Control Study. Clin. Cancer Res..

[111] Pietrantonio F., Caporale M., Morano F., Scartozzi M., Gloghini A., De Vita F., Giommoni E., Fornaro L., Aprile G., Melisi D. (2016). HER2 Loss in HER2-positive Gastric or Gastroesophageal Cancer After Trastuzumab Therapy: Implication for Further Clinical Research. Int. J. Cancer.

[112] Zhao D., Samuel J., Klempner S.J., Chao J. (2019). Progress and Challenges in HER2-positive Gastroesophageal Adenocarcinoma. J. Hematol. Oncol..

[113] Battaglin F., Naseem M., Puccini A., Lenz H.J. (2018). Molecular biomarkers in gastro-esophageal cancer: Recent developments, current trends and future directions. Cancer Cell Int..

[114] Cho J., Ahn S., Son D.S., Kim N.K., Lee K.W., Kim S., Lee J., Park S.H., Park J.O., Kang W.K. (2019). Bridging genomics and phenomics of gastric carcinoma. Int. J. Cancer.

[115] Kim C., Lee C.K., Chon H.J., Kim J.H., Park H.S., Heo S.J., Kim H.J., Kim T.S., Kwon W.S., Chung H.C. (2017). PTEN loss and level of HER2 amplification is associated with trastuzumab resistance and prognosis in HER2-positive gastric cancer. Oncotarget.

[116] Janjigian Y.Y., Sanchez-Vega F., Jonsson P., Chatila W.K., Hechtman J.F., Ku G.Y., Riches J.C., Tuvy Y., Kundra R., Bouvier N. (2018). Genetic predictors of response to systemic therapy in esophagogastric cancer. Cancer Discov..

[117] Pectasides E., Stachler M.D., Derks S., Liu Y., Maron S., Islam M., Alpert L., Kwak H., Kindler H., Polite B. (2018). Genomic heterogeneity as a barrier to precision medicine in gastroesophageal adenocarcinoma. Cancer Discov..

[118] Gao J., Wang H., Zang W., Li B., Rao G., Li L., Yu Y., Li Z., Dong B., Lu Z. (2017). Circulating tumor DNA functions as an alternative for tissue to overcome tumor heterogeneity in advanced gastric cancer. Cancer Sci..

[119] Kankeu Fonkoua L., Yee N.S. (2018). Molecular Characterization of Gastric Carcinoma: Therapeutic Implications for Biomarkers and Targets. Biomedicines.

